# Continuing Orthohantavirus Circulation in Deer Mice in Western Montana

**DOI:** 10.3390/v13061006

**Published:** 2021-05-27

**Authors:** Brandi N. Williamson, Kimberly Meade-White, Kristin Boardman, Jonathan E. Schulz, Carson T. Telford, Dania M. Figueroa Acosta, Trenton Bushmaker, Robert J. Fischer, Kyle Rosenke, Heinz Feldmann

**Affiliations:** Laboratory of Virology, Division of Intramural Research, National Institute of Allergy and Infectious Diseases, National Institutes of Health, Hamilton, MT 59840, USA; kmeade-white@niaid.nih.gov (K.M.-W.); kristinboardman94@gmail.com (K.B.); jonathan.schulz@nih.gov (J.E.S.); carson.ted.telford@emory.edu (C.T.T.); dania.figueroa@icahn.mssm.edu (D.M.F.A.); bushmakertj@niaid.nih.gov (T.B.); fischerro@niaid.nih.gov (R.J.F.); kyle.rosenke@nih.gov (K.R.); feldmannh@niaid.nih.gov (H.F.)

**Keywords:** *Sin Nombre orthohantavirus*, *Peromyscus maniculatus*, lung, genome detection, Bitterroot Valley, Montana

## Abstract

Hantavirus pulmonary syndrome (HPS) is an often-fatal disease caused by New World hantaviruses, such as *Sin Nombre orthohantavirus* (SNV). In the US, >800 cases of HPS have been confirmed since it was first discovered in 1993, of which 43 were reported from the state of Montana. The primary cause of HPS in the US is SNV, which is primarily found in the reservoir host *Peromyscus maniculatus* (deer mouse). The reservoir host covers most of the US, including Montana, where multiple studies found SNV in local deer mouse populations. This study aimed to check the prevalence of SNV in the deer mice at popular recreation sites throughout the Bitterroot Valley in Western Montana as compared to previous studies in western Montana. We found high prevalence (up to 20%) of deer mice positive for SNV RNA in the lungs. We were unable to obtain a SNV tissue culture isolate from the lungs but could passage SNV from lung tissue into naïve deer mice. Our findings demonstrate continuing circulation of SNV in western Montana.

## 1. Introduction

Hantaviruses are rodent-borne, tri-segmented RNA viruses in the genus *Orthohantavirus*, family *Hantaviridae*, order *Bunyavirales* [1,2]. Historically they have been classified as either New or Old World hantaviruses [2]. Old World hantaviruses, such as *Hantaan*, *Seoul*, and *Dobrava-Belgrade orthohantavirus*, cause hemorrhagic fever with renal syndrome (HFRS), whereas New World hantaviruses, such as *Andes* and *Sin Nombre orthohantavirus* (SNV), cause hantavirus (cardio)pulmonary syndrome (HPS or HCPS) [2]. HPS was first described during the 1993 outbreak of the then emerging SNV in the Four Corners region of the southwestern United States [3]. HPS presented as an acute respiratory disease that resulted in rapid progression to death in an alarmingly high percentage of previously healthy young adults [3]. Within the year, the deer mouse, *Peromyscus maniculatus*, was identified as the primary rodent reservoir for SNV [4]. It was later determined that cases of HPS caused by SNV had occurred as early as 1959 [5]. Currently, >800 HPS cases have been reported in the US (CDC Viral Special Pathogens Unit, personal communication), the majority believed to be caused by SNV [6].

Humans are exposed to hantaviruses in peridomestic settings where deer mice excrete urine and feces that may then be aerosolized by human activity [7]. Human cases peak during the spring and early summer months [8]. Though not clearly understood, hypotheses abound for the seasonality of cases, the timing, and the rarity of human infection [7].

Following the outbreak of HPS cases, field studies were conducted throughout the United States and other regions, including Montana [6,9,10,11]. A substantial number of field studies examined the population dynamics of, as well as SNV prevalence in, the small mammal population in western Montana. A number of these field sites, adjacent to but not including the Bitterroot Valley, were annually sampled from the mid-1990s through 2013 [12,13,14]. Douglass et al. found an overall prevalence of antibodies to SNV in 15.8% of the deer mice tested from 18 study sites in Montana between 1994–1999 [9]. From 1996–1999, three of those study sites contained deer mice with a combined SNV antibody prevalence of 24.5%. The site with the highest prevalence was near Butte, Montana with 28.8% [10]. A related Montana field study looked for the presence of SNV RNA and antibodies in the blood of deer mice [14]. Of the antibody positive deer mice blood samples tested, 19% were PCR positive for SNV [14].

The goal of this study was to survey the prevalence of hantaviruses within the Bitterroot Valley of western Montana and to provide data for public health risk assessment in a popular recreational region in western Montana.

## 2. Materials and Methods

### 2.1. Biosafety

All tissue culture and animal work with SNV was performed in the biosafety level-3 and -4 (BSL3 and BSL4) facilities at the Rocky Mountain Laboratories (RML), Division of Intramural Research, NIAID, NIH. Work followed standard operating procedures approved by the Institutional Biosafety Committee. Animal work was approved by the RML Animal Care and Use Committee (ACUC) and performed following institutional and national regulations and laws. Animal work in the field was performed near the site of capture away from people. During animal processing, researchers wore Power Air Purifying Respirators (3M, St. Paul, MN, USA), Tyvek suits, and double gloves. Equipment was decontaminated by either a 70% ethanol or 10% bleach solution. Traps were disinfected in 10% bleach solution. After completion of the work, researchers sprayed each other down with a 10% bleach solution before doffing personal protective equipment (PPE). All waste generated in the field was inactivated with 10% bleach and transported back to the laboratory and incinerated immediately.

### 2.2. Field Locations

**Figure 1:**
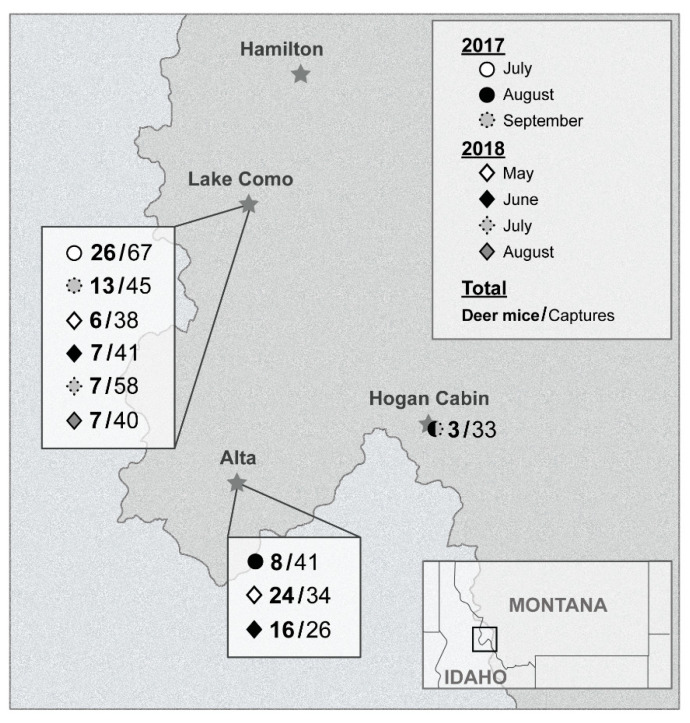
Map of field sites and capture data for 2017 and 2018 trapping seasons. The number of deer mice per total number animal capture is listed next to symbols for the trapping month (May, June, July, or August) per field site (Lake Como, Alta, or Hogan Cabin).

Four different locations were targeted over two years (2017 and 2018) (Figure 1). In 2017, small mammals were captured in and around two adjacent cabins, Hiker’s cabin (16–18 July) (46°03′56″ N 114°14′43″ W) and Wood’s cabin (6–8 September) (46°03′55″ N 114°14′40″ W) at Lake Como, Ravalli County, Montana. Two other locations were targeted in 2017, a private property near Alta, Montana (1–3 August) (45°37′09″ N 114°18′01″ W) and a forest service cabin near Lost Trail Pass, Montana called Hogan cabin (30 August–1 September) (45°42′49″ N 113°52′47″ W). The following summer, 2018, only two locations were targeted from the previous summer: Alta and Lake Como. The Alta site was visited twice, 14–16 May and 4–6 June. Trapping at Lake Como occurred 23–25 May, 20–22 June, 25–27 July, and 22–24 August. During the second summer, no traps were set inside either Hiker’s or Wood’s cabin at Lake Como.

### 2.3. Animal Trapping

Small mammals were live trapped with Sherman traps (H.B. Sherman Live Traps Inc., Tallahassee, FL, USA) and, in 2017, Tomahawk traps (Tomahawk Live Trap, Hazelhurst, WI, USA). Between 60–100 traps were set in the afternoon or evenings (depending on location). Traps were set in and around structures, ponds, trees, rocks, and stumps, and were baited with crushed oats. Captured animals were anesthetized with isoflurane, then weighed and measured. Upon request by the private landowners, we euthanized every animal captured on their property. At other locations, only those captured in the cabins or up to 15% of deer mice (permitted by two RML ACUC approved Animal Study Protocols: 2015-011 and 2018-014-F and two Montana Fish Wildlife and Parks Scientific Collectors Permits: 2017-106-W and 2018-048-W) were euthanized and necropsied. Blood was collected by cheek bleed for catch and release animals, as well as by cardiac puncture for those to be euthanized. Up to 140 µL of blood was added to the AVL buffer (QIAGEN, Germantown, MD, USA) and placed on dry ice. The remainder was put in serum separator tubes (SST) (Sarstedt, Newton, NC, USA) and placed on ice packs. An 8 mm diameter-sized portion of the heart, lung, kidney, spleen, and liver were collected from necropsied animals. During the 2018 trapping season, urogenital, oral, and rectal swabs were collected and put into 1 mL of Viral Transport Medium (VTM), a modified Eagle’s cell culture medium (MEM) containing 8% Hank’s salt solution, 192 units/mL of penicillin, 192 µg/mL of streptomycin, 92 units/mL polymyxin B sulfate, 48 units/mL nystatin, 48 µg/mL of gentamicin, and 9.6% glycerol. All live animals were released at site of capture.

### 2.4. Sample Processing

At the laboratory, the SST tubes were spun, serum collected, and gamma irradiated with 8 MRads [15]. RNA was extracted from the AVL blood samples and swabs using the QIAamp Viral RNA Mini kit according to the manufacturer’s protocol (QIAGEN, Germantown, MD, USA). Tissue samples were processed using the RNeasy Mini kit and the TissueLyser II according to the manufacturer’s animal tissues protocol (QIAGEN, Germantown, MD, USA).

### 2.5. qRT-PCR

Viral RNA was detected using the QuantiFast Probe RT-PCR Kit and the Rotor-Gene 3000 according to the manufacturer’s instructions. The primers were SNV-S179F: 5′-GCAGACGGGCAGCTGTG–3′ and SNV-S245R: 5′–AGATCAGCCAGTTCCCGCT–3′ with the probe 5′-6FAM-TGCATTGGAGACCAAACTCGGAGAACTT-BBQ-3′. Cycling was as follows: 10 min at 50 °C, 5 min at 95 °C and 40 cycles of 95 °C for 15 s and 58 °C for 21 s.

### 2.6. Serology

Serology was tested with slight modifications to the previously published field ELISA [16]. Briefly, the plates were coated with 200 ng of the truncated SNV nucleocapsid protein and IgG was detected using recombinant Protein A/G (ThermoFisher Scientific, Waltham, MA, USA). Plates were read on a Microplate reader at 405 nm (BioTek, USA). Samples were considered positive if they were 3× the standard deviation plus the mean of the negative controls at a dilution ≥1:400.

### 2.7. Sequencing and Phylogenetic Analysis

L, M, and S segments from qRT-PCR positive samples were amplified and sequenced using the primers in Table S1. Using Superscript III reverse transcriptase (ThermoFisher Scientific, Waltham, MA, USA) and IProof DNA Polymerase (Bio-Rad, Hercules, CA, USA) according to the manufacturer’s instructions, amplicons of each segment were made. The PCR products of the corresponding sizes were excised and purified using the NucleoSpin Gel and PCR Clean-up Kit (Macherey-Nagel, Bethlehem, PA, USA), then sent for Sanger sequencing at the RML Research Technologies Branch Genomic Core using the primers listed in Table S1. Phylogenetic analysis was done using Geneious Prime 11 2019.0.4 (Biomatters Ltd., San Diego, CA, USA). Analysis was performed using available sequences on GenBank matching the regions we sequenced. Reassortment analysis was done through manual comparison of the phylogenetic trees.

### 2.8. Virus Isolation In Vitro and In Vivo

The lungs of four deer mice, which were found to be positive using qRT-PCR, were homogenized in Dulbecco’s modified eagle’s medium (DMEM) (MilliporeSigma, St. Louis, MO, USA) using the TissueLyser II and one 5 mm stainless steel bead (QIAGEN, Germantown, MD, USA). The samples were further diluted 1:100 and 1:1000 in additional DMEM. The two lung homogenates with the lowest Ct values by qRT-PCR, SNV BV85, and 121 were selected for in vitro isolation attempts. The diluted lung homogenates were incubated on Vero E6 cells for 1 h at 37 °C. After 1 h, the infection medium was removed and replaced with DMEM with 2% FBS. As a control, the SNV isolate, SNV77734, was grown on Vero E6 cells. Supernatant samples were taken on days 3, 9, and 15. The same RNA extraction and qRT-PCR protocols were followed as outlined above for the field blood and swab samples. For the in vivo isolation attempt, we utilized an in-house deer mouse colony (RML ACUC approved Animal Study Protocol: 2017-039). Three SNV positive deer mice lungs (SNV BV121, 213, and 254) were used to inject i.m. and i.p., six deer mice with 200 µL of either a 1:100 or 1:1000 dilution of each lung homogenate. The deer mice were bled and tested via qRT-PCR and ELISA to find the presence of RNA or antibody to SNV at days 12, 20, 28, and 60 (terminal). Lungs were harvested at day 60, then 30 mg of tissue was homogenized in an RLT buffer and processed through the animal tissues protocol of the RNeasy Extraction kit (QIAGEN, Germantown, MD, USA). This RNA was run through the qRT-PCR protocol listed above. One mouse seroconverted by day 28 and was positive for RNA in the lung at day 60. Homogenate of this lung was diluted 1:100 in DMEM and injected into 10 additional deer mice. Lungs were harvested at day 60 post infection and processed the same way as the first passage lung samples.

## 3. Results

### 3.1. Ecological Investigation

In the summer of 2017, a total of 11,347 trap-hours and 565 trap-nights were totaled. We caught a total of 186 animals, 50 of which were deer mice. In the summer of 2018, a total of 21,856 trap-hours and 1175 trap-nights were totaled. We caught a total of 237 animals, 67 of those were deer mice. By location, no more than 26 deer mice were caught in a single trapping session (Figure 1).

### 3.2. Molecular Investigation

Over the course of both summers, 3.6% of the deer mice (4 of 112 tested) had detectable RNA circulating in the blood and 18.9% of the deer mice (14 of 74 tested) had detectable SNV RNA in their lungs (Table 1). Blood samples for 221 non-deer mouse species were tested using qRT-PCR and none were found to be positive (data not shown). Overall, 12.7% of the deer mice (13/102) had antibodies against SNV. At Lake Como, over the course of both seasons, 43 deer mice were captured but only a small percentage were positive for SNV RNA in their lungs (8.7%) or by serology (7.7%). At Alta, over the course of two trapping seasons, a high percentage (22.9%) of captured deer mice had detectable SNV RNA in the lungs. In all locations, the percent of seropositive deer mice was lower than the percentage of SNV RNA positive mice (Table 1). Of the 14 SNV RNA lung-positive deer mice, 12 were males (85.7%), compared 65% of the overall capture being males (76/117).

During the 2018 season, rectal, urogenital, and oral swabs were taken from all of the deer mice captured. The swabs from the 11 mice positive for SNV RNA in the lungs were tested by qRT-PCR and no detectable RNA was found.

### 3.3. Phylogenetic Analysis

**Figure 2:**
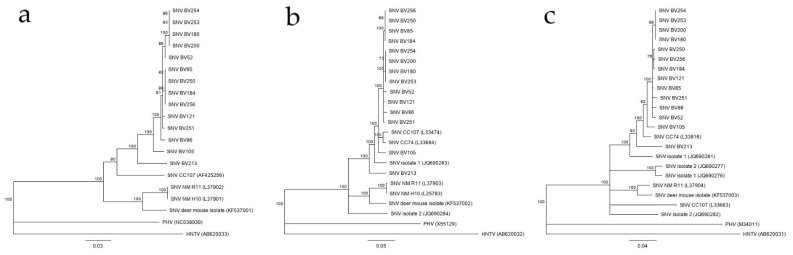
(**a**) Phylogenetic tree of 1121 bp of the L segment, (**b**) tree of 2088 bp of the M segment, and (**c**) tree of 1641 bp of the S segment of Bitterroot Valley *Sin Nombre orthohantavirus* (SNV) sequences compared to other SNV sequences throughout the western United States. The trees were inferred using the neighbor-joining method based on the Tamura-Nei model. Bootstrap percentages (1000 replicates) above 70% are shown next to the branches. The branch lengths are the number of base substitutions per site. GenBank submission numbers are listed in parentheses. Analyses were conducted in Geneious Prime 11. HNTV = Hantaan virus, PHV = Prospect Hill virus.

Phylogenetic comparisons were done with 1488 bp for the S segment, 2021 bp for the M segment and 1121 bp for the L segment (Figure 2). The sequence for each segment (S, M, and L) of the 14 local SNV strains (BV52 through BV256) were submitted to GenBank (S segment: MW177646-MW177660, M segment: MW177661-MW177673, L segment: MW177674-177687). In the S segment tree, the Bitterroot Valley strains were grouped closest with the CC74 strain (GenBank accession L33816) from northern California rather than with the other Montana strains (GenBank accessions JQ690281, JQ690282, JQ690276, and JQ690277) and New Mexico strains (GenBank accessions L37904, KF537003) (Figure 2a) [17,18]. The S segment of all SNV strains compared in the tree, are within 80% nucleotide identity, which increased to 87% when comparing only the coding region of the S segment. The Bitterroot Valley strains were all within 87% nucleotide identity in the S segment alignment. In the M segment tree, both CC74 and CC107 sequences (GenBank accession L33684 and L33474, respectively) were grouped closely with the Bitterroot Valley strains, along with the SNV isolate 1 (GenBank accession JQ690283) from Gregson, MT (Figure 2b) [17]. All SNV sequences in the M segment tree were within 87% nucleotide identity and all Bitterroot Valley SNV strains were within 94% nucleotide identity of each other. In the L segment tree, all the Bitterroot Valley SNV strains formed in a monophyletic group and strain CC107 (GenBank accession AF425256) was the next closest related virus (Figure 2c). All Bitterroot Valley SNV strains in the L segment tree were within 93% nucleotide identity of each other. The amino acid identity was above 98% in each segment comparison. SNV BV213 was consistently the most distantly related Bitterroot Valley strain in all three segments (Figure 2).

The phylogenetic analysis was suggestive of reassortment for two Bitterroot Valley SNV strains, BV52 and 85. BV52 grouped most closely with BV86, 121, and 251 in the S and M segment trees but grouped closer to SNV BV180, 200, 253, and 254 in the L segment tree. BV85 grouped closely with BV184, 250, and 256 in the M and L segment trees but was less closely related with that clade in the S segment tree. There were not enough sequences for all segments of SNV strains outside of the Bitterroot Valley to perform reassortment analysis on a wider geographical scale.

### 3.4. Virus Isolation

During the in vitro isolation attempt, the viral load for the field caught SNV-positive deer mice lung samples decreased over time until they were undetectable by day 15 indicating failure in virus isolation. In contrast, the SNV77734 lung samples, serving as a positive control for virus isolation, increased in viral load for each timepoint, as measured by qRT-PCR. In the first passage of field SNV-positive lung tissue into the deer mouse laboratory colony, one mouse (infected with the 1:100 dilution of SNV BV254 lung tissue) was positive in the lungs by qRT-PCR at day 60 post infection with 5.11 × 10^4^ genome copies/mg of tissue. After the second passage into 10 additional aged (>15 weeks) male deer mice, two mice were positive in the lungs by qRT-PCR at day 60 with 7.78 × 10^3^ and 6.56 × 10^4^ genome copies/mg of tissue.

## 4. Discussion

The prevalence of circulating SNV in the deer mouse population of the Bitterroot Valley is currently at similar levels as found in previous studies in western Montana two decades ago [9,10,14]. This finding remains of public health interest but is not surprising given many surrounding counties in western Montana have reported evidence of SNV in deer mice or have had locally acquired HPS cases in the past [9,10,14,18]. A high number of SNV RNA positive deer mice were found within specific sites in the Bitterroot Valley, most specifically the Alta area with more than 20% of the mice actively positive for SNV RNA (Table 1). SNV infection prevalence has been proposed to be highest in the spring when the population structure is skewed toward older mice [19]. In our study, the majority of capture dates were late spring to early summer suggesting that SNV prevalence in the Bitterroot Valley is likely even higher in early spring, were this hypothesis true.

All sequences derived from SNV captured in the Bitterroot Valley were most closely related to either the CC74 or the CC107 SNV isolate from East Central California and previous Montana SNV isolates and less related to the New Mexico isolates in the phylogenetic analysis (Figure 2). While two possible reassortment events were observed in the BV52 and 85 strains, a phylogenetic analysis with the full genome sequence of all the segments would need to be done to determine if these were true reassortment events or artifacts from comparing only a portion of each segment. The Bitterroot Valley SNV strains are distinct with mutations occurring in all segments but a deeper dive into the SNV genomic variation by in vitro and in vivo studies would be needed to decipher if these hantaviruses represent any distinct biological phenotype. For this, virus isolates would help to perform growth kinetics in tissue culture and to evaluate virulence and transmissibility in animal models such as the hamster SNV infection model [20] or the SNV nonhuman primate disease model [21]. Unfortunately, hantaviruses are difficult to isolate in tissue culture which also failed in our attempts. However, we were successful in growing SNV in deer mice from field material and virus stocks could be prepared from deer mouse lung tissue for future inoculation studies [21]. Thus, having an in-house colony of the reservoir species provides a handy tool for virus biology, pathogenesis, and transmission studies.

Although SNV is highly prevalent in the deer mouse populations in western Montana, HPS remains a rare disease. Locally in the Bitterroot Valley, deer mice have comparable prevalence of SNV as other studies have found in the state of Montana over the last two decades. The mechanisms and reasons for this are poorly understood. Even in high-risk groups in Nebraska, California, Texas, and New Mexico, such as field workers and forest workers, the exposure to SNV is limited with seroprevalences of <1% [22,23,24]. Understanding the hantavirus dynamics in the reservoir host population and comparisons between areas with high and low transmission risk might help elucidate answers to these questions regarding SNV and other hantaviruses.

In conclusion, our study revealed continued prevalence (up to 20%) of SNV in deer mice in western Montana. SNVs from the Bitterroot Valley were found to be most closely related to the east-central region in California isolates with possible evidence for reassortment. Here, we demonstrated an alternative hantavirus isolation method by utilizing an in-house deer mouse colony. Continuing ecological studies are needed to monitor SNV prevalence for public health preparedness and response.

## Figures and Tables

**Table 1:** SNV qRT-PCR and serology results for deer mice by location and date.

Location	Year	Blood RT-PCR		Lung RT-PCR		Serology	
		No. Positive/No. Tested	% Positive	No. Positive/No. Tested	% Positive	No. Positive/No. Tested	% Positive
Lake Como	2017	0/39	0	1/20	5	1/37	2.7
Alta		2/8	25	3/8	37.5	2/8	25
Hogan Cabin		1/3	33.3	1/3	33.3	0/3	0
2017 Total		3/50	6	5/31	16.1	3/48	6.3
Lake Como	2018	1/22	4.5	1/3	33.3	3/15	20
Alta		0/40	0	8/40	20	7/39	17.9
2018 Total		1/62	1.6	9/43	20.9	10/54	18.5
Total		4/112	3.6	14/74	18.9	13/102	12.7

## Data Availability

Data is publicly available on FigShare at https://figshare.com/articles/dataset/Continuing_orthohantavirus_circulation_in_deer_mice_in_Western_Montana/14390705. Accessed: 15 April 2021.

## Supplementary Materials

The following are available online at https://www.mdpi.com/article/10.3390/v13061006/s1, Table S1: Amplification and sequencing primers.
