# Supplementary material for: Continuing Orthohantavirus Circulation in Deer Mice in Western Montana
Source: Viruses. 2021 May 27;13(6):1006. doi: 10.3390/v13061006 (PMC8226622; doi:10.3390/v13061006)
Supplement: Supplementary file 1 [file viruses-13-01006-s001.zip › viruses-1204953-supplementary.pdf]

Table S1. Amplification and sequencing primers. Amplification primers are in bold, the other primers are internal sequencing primers for the amplicon.

Segment	Primer Name	Primer Sequence (5′ to 3′)	Expected Amplicon Size (bp)
L	**SNVLF**	**CAGACAAAGCAGCACAAAGCTCAAGC**	**1240**
	**SNVLR**	**GCCATACCTATACCTGCTGTGG**	
	SNVF2	TCATCGCTATATAGATTCTATGGATGAGC	
	SNVR2	GCATTTGTAGGAGAAACTGTTGTC	
	SNVL3116F	CTTCATCGCTATATAGATTCTATGGA	
	SNVL3665F	GCTGCTGCACARAGTAGATGTGT	
M	**SNVM472F**	**GCCTGTAATCAAACACATTGTCTTC**	**2049**
	**SNVM2521R**	**AGGTTTGTTCTGTCCCCAATTGAAT**	
	SNVM979R	GCCAAGGTACCATCTACAGAAAC	
	SNVM1037R	GGTGCCCCAGCAAATGCAA	
	SNVM1459F	TCACTGATCCCTGGAGTTGC	
	SNVM1526R	GCCCAGCCATGAAGACCTG	
	SNVM1899F	TCCAGAAGTAAGGAAAGGTTGTTA	
	SNVM2029R	CACCATGTGCTGTATCTGACCA	
S	**SNVS488F**	**TYAARGAAAACAAGGGRACAAGAAT**	**1559**
	**SNVS2047R**	**GTTGAGGTAATAGGGAAGGGGATA**	
	SNVS930F	TGCCACTATATTCGCAGATATTGC	
	SNVS595R	GAGCAGTWGGCAWAGAAACATACAG	
	SNVS1021R	CACATATAATGCTGTVGGTGGACA	
	SNVS1419F	CTAGGGTGGGTTYAAGGGCCAA	
	SNVS1557R	ACTTAACAAAACTTAACAGGATTTAATA	
